# Supplementary material for: Cold Atmospheric Plasma-Treated PBS Eliminates Immunosuppressive Pancreatic Stellate Cells and Induces Immunogenic Cell Death of Pancreatic Cancer Cells
Source: Cancers (Basel). 2019 Oct 19;11(10):1597. doi: 10.3390/cancers11101597 (PMC6826654; doi:10.3390/cancers11101597)
Supplement: Supplementary file 1 [file cancers-11-01597-s001.pdf]

Supplementary Materials: Cold Atmospheric Plasma-Treated PBS Eliminates Immunosuppressive Pancreatic Stellate Cells and Induces Immunogenic Cell Death of Pancreatic Cancer Cells

Jinthe Van Loenhout, Tal Flieswasser, Laurie Freire Boullosa, Jorrit De Waele, Jonas Van Audenaerde, Elly Marcq, Julie Jacobs, Abraham Lin, Eva Lion, Heleen Dewitte, Marc Peeters, Sylvia Dewilde, Filip Lardon, Annemie Bogaerts, Christophe Deben and Evelien Smits

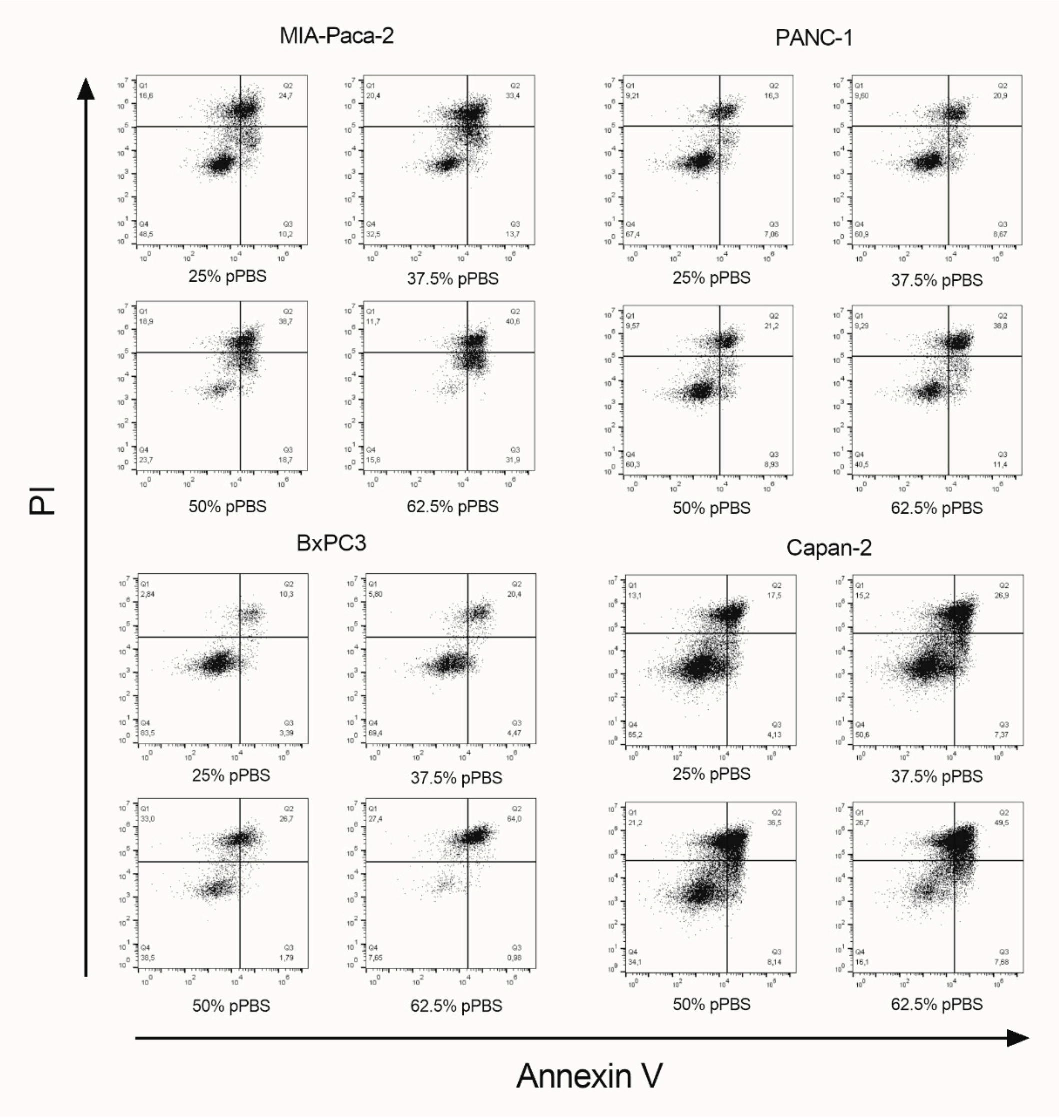

Figure S1. Dot plots showing the flow cytometric analysis of Annexin V and PI staining after 25%, 37.5%, 50% and 62.5% pPBS treatment in all PCC lines. Q1 = AnnV-/PI+; Q2 = AnnV+/PI+; Q3 = AnnV-/PI-; Q4 = AnnV+/PI-.

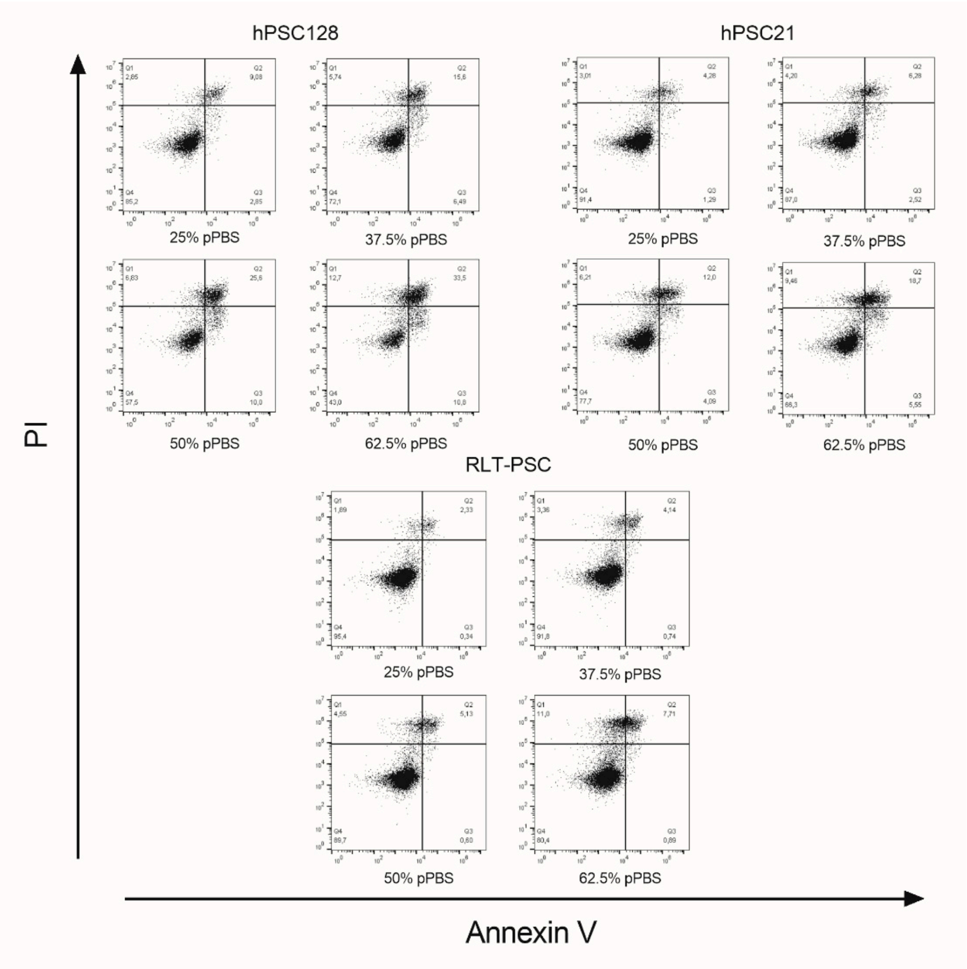

Figure S2. Dot plots showing the flow cytometric analysis of Annexin V and PI staining after 25%, 37.5%, 50% and 62.5% pPBS treatment in all PSC lines. Q1 = AnnV-/PI+; Q2 = AnnV+/PI+; Q3 = AnnV-/PI-; Q4 = AnnV+/PI-.

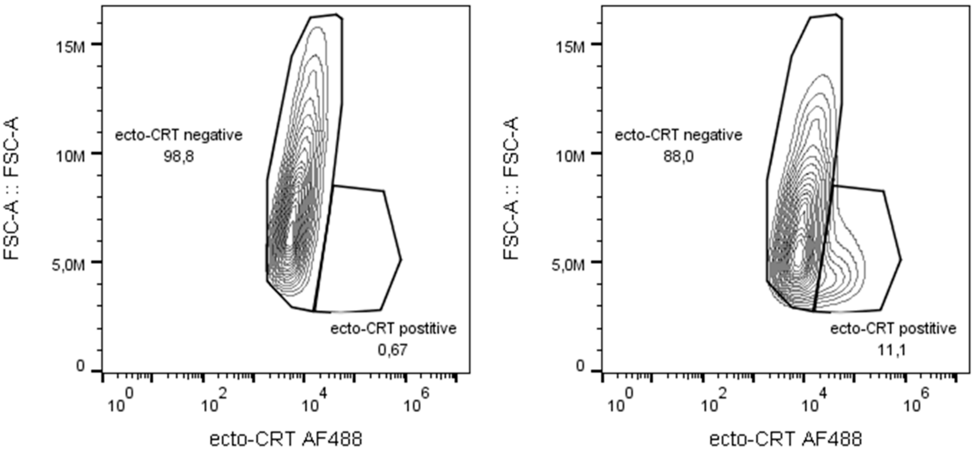

Figure S3. Gating strategy of surface exposure of ecto-CRT. Contour plots showing the flow cytometric analysis of ecto-CRT staining after 48h of 50% pPBS treatment (left) compared to untreated condition (right) in the BxPC3 cell line (PI- cells) showing the differences in Δ MFI (MFI treated vs MFI untreated).

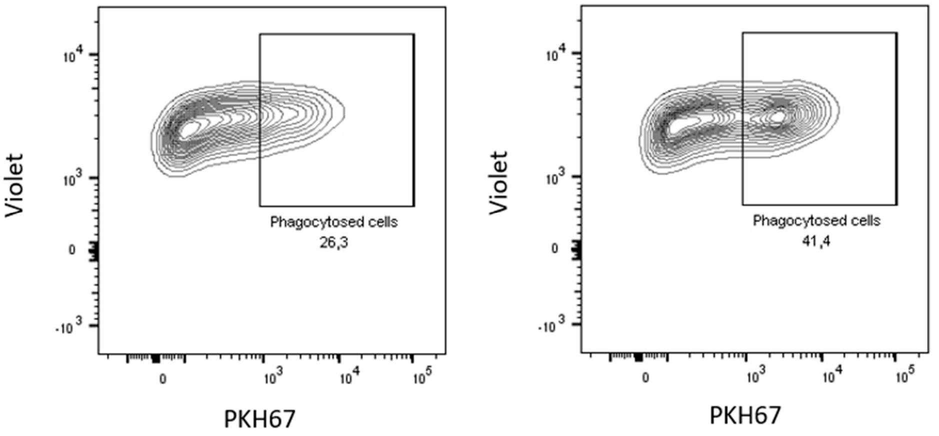

Figure S4. Gating strategy of phagocytosis. Contour plots showing flow cytometric analysis of percentage phagocytosis (left: MIA-Paca-2, untreated; right: MIA-Paca-2, 50% pPBS treatment). Target cells labelled with PKH67 dye and DC labeled with CellTracker Violet BMQC dye are cocultured for 48h (E:T ratio, 1:1). Phagocytosis of the PKH67+ target cells by violet labeled DC is expressed as the %violet+PKH67+ cells within the violet+ DC population.

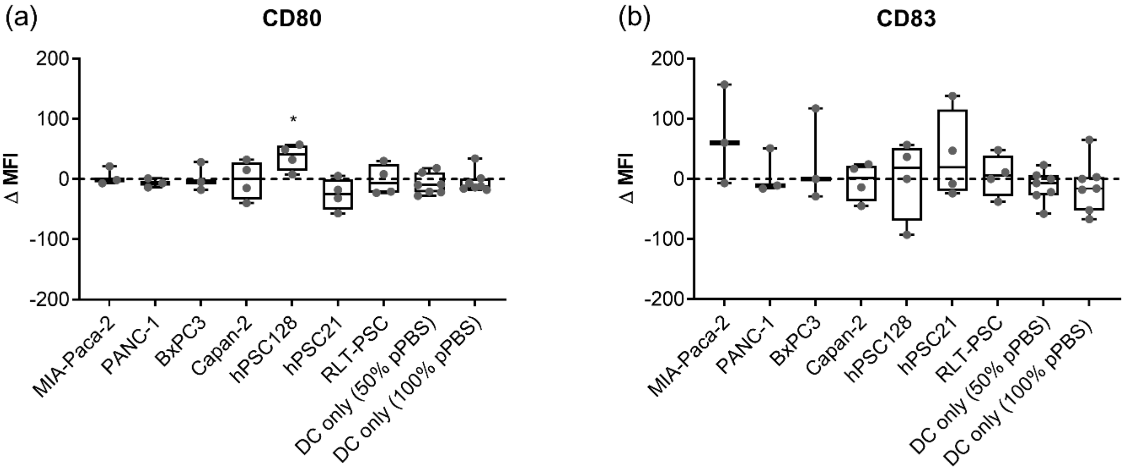

Figure S5. CD80 and CD83 expression on DC after coculture with pPBS-treated PSC and PCC. (**a**) Boxplot from minimum to maximum value of Δ MFI of the maturation marker CD80. (**b**) Boxplot from minimum to maximum value of Δ MFI of the maturation marker CD83. CD80 and CD83 expression is examined on immature DC after 48h of coculture of pPBS-treated PCC and PSC (E:T ratio, 1:1) and after pPBS treatment on immature DC without coculture using flow cytometry. Δ MFI represents [(MFI staining treated – MFI isotype treated) – (MFI staining untreated – MFI isotype untreated)]. Treatment of 50% pPBS is used for MIA-Paca-2 and Capan-2, treatment of 100% pPBS is used for PANC-1, BxPC3, hPSC128, hPSC21 and RLT-PSC. Every dot represents a different healthy donor with ≥3 donors used per cell line. $p<0.05$ significant differences compared to untreated control (*).

© 2019 by the authors. Licensee MDPI, Basel, Switzerland. This article is an open access article distributed under the terms and conditions of the Creative Commons Attribution (CC BY) license (http://creativecommons.org/licenses/by/4.0/).
